# The Differential Effects of Caregiving Intensity on Overnight Hospitalization in the Previous 2 Years

**DOI:** 10.1093/geroni/igaa057.249

**Published:** 2020-12-16

**Authors:** Kylie Meyer, Zachary Gassoumis, Kathleen Wilber

**Affiliations:** 1 UT Health Sciences at San Antonio, San Antonio, Texas, United States; 2 University of Southern California, Los Angeles, California, United States

## Abstract

Caregiving for a spouse is considered a major stressor many Americans will encounter during their lifetimes. Although most studies indicate caregiving is associated with experiencing diminished health outcomes, little is known about how this role affects caregivers’ use of acute health services. To understand how spousal caregiving affects the use of acute health services, we use data from the Health and Retirement Study. We apply fixed effects (FE) logistic regression models to examine odds of experiencing an overnight hospitalization in the previous two years according to caregiving status, intensity, and changes in caregiving status and intensity. Models controlled for caregiver gender, age, race, ethnicity, educational attainment, health insurance status, the number of household residents, and self-assessed health. Overall, caregivers were no more likely to experience an overnight hospitalization compared to non-caregivers (OR 0.92; CI 0.84 to 1.00; p-value=0.057). However, effects varied according to the intensity of caregiving and the time spent in this role. Compared to non-caregivers, for example, spouses who provided care to someone with no need for assistance with activities of daily living had lower odds of experiencing a hospitalization (OR 0.77; CI 0.66 to 0.89). In contrast, caregivers who provided care to someone with dementia for 4 to <6 years had 3.29 times the odds of experiencing an overnight hospitalization (CI 1.04 to 10.38; p-value=0.042). Findings indicate that, although caregivers overall appear to use acute health services about as much as non-caregivers, large differences exist between caregivers. Results emphasize the importance of recognizing diversity within caregiving experiences.

